# ART3 regulates triple-negative breast cancer cell function via activation of Akt and ERK pathways

**DOI:** 10.18632/oncotarget.10306

**Published:** 2016-06-27

**Authors:** Ling Tan, Xiaodan Song, Xin Sun, Ning Wang, Ying Qu, Zhijun Sun

**Affiliations:** ^1^ Department of Breast, Pancreas, and Thyroid Surgery, Second Affiliated Hospital of Chongqing Medical University, Chongqing, China; ^2^ Department of Bioengineering, College of Engineering, Northeastern University, Boston, MA, USA; ^3^ Department of Surgery, Department of Obstetrics and Gynecology, Women's Cancer Program Samuel Oschin Comprehensive Cancer Institute, Cedars-Sinai Medical Center, Los Angeles, CA, USA

**Keywords:** Akt, ART3, ERK, triple-negative breast cancer

## Abstract

Triple-negative breast cancers (TNBCs) are defined by lack of expressions of estrogen, progesterone, and ERBB2 receptors. Because biology of TNBC is poorly understood, no targeted therapy has been developed for this breast cancer subtype and chemotherapy is its only systemic treatment modality. In this study, we firstly determined that the expression of human ecto-ADP-ribosyltransferase 3 (ART3) is significantly associated with the basal-like breast cancer subgroup, which is largely overlapped with TNBC, through analyzing published data sets. We also found that ART3 protein is significantly overexpressed in human TNBC tumors tissue and cell lines through using immunohistochemistry and immunoblotting. Overexpression of ART3 in MDA-MB-231 breast cancer cells increased cell proliferation, invasion, and survival in vitro and growth of xenograft tumors. Conversely, knockdown of ART3 in breast cancer cells inhibited cell proliferation and invasion. In addition, we showed that ART 3 overexpression activated AKT and ERK in vitro and in xenograft tumors. Together, our findings demonstrate that ART3 is a critical TNBC marker with functional significance.

## INTRODUCTION

The process of ecto-ADP-ribosylation, catalyzed by ecto-ADP-ribosyltransferase (ART), is a critical post-translational modification of proteins [1–6]. ART3 expression has been found to be related to physiological and pathological functions including quantitative impairment of spermatogenesis [7] and myocardial cells [8]. However, the function of ART3 in tumor cells is unclear. To date, there is only one report in this regard, indicating that ART3 might increase the tumor size of hepatocellular carcinoma (HCC) with loss of heterozygosity (LOH) [9].

Triple-negative breast cancers (TNBCs) are defined by lack of expressions of estrogen, progesterone, and ERBB2 receptors (known as ER, PR, and HER2 respectively). As the most malignant subtype, TNBC accounts for 10% to 20% of breast cancer cases and is characterized by frequent distant metastasis and local recurrence, as well as poor prognosis [10, 11]. Currently, chemotherapy is the only systemic treatment option for TNBC. Previous studies indicated several possible treatments for TNBC, including EGFR-targeted drugs (such as cituximab, gefitinib, and erlotinib) [12], anti-VEGF or -VEGFR drugs (such as bevacizumab and sorafenib) [13, 14], PARP-1 inhibitors (olaparib and iniparib), Src inhibitors, a survivin suppressant [15], et al. However, these clinical trials have not yielded promising or conclusive results, probably due to the fact that these target proteins may not be essential for TNBC tumor growth. Therefore, there is an urgent need to identify specific molecular targets for TNBC treatment.

In this study, we found that ART3 mRNA was overexpressed in human TNBC compared with non-TNBC and correlated with a shorter survival for breast cancer patients. We also showed the association of ART3 protein levels with TNBC using an immunohistochemical assay in human breast cancer specimens. In addition, we found that ART3 induces TNBC cell growth and invasion by activating Akt and ERK. Taken together, this study provides the first evidence that ART3 serves as a critical marker for TNBC.

## RESULTS

### ART3 is specifically overexpressed in basal-like breast cancers

**Figure 1:**
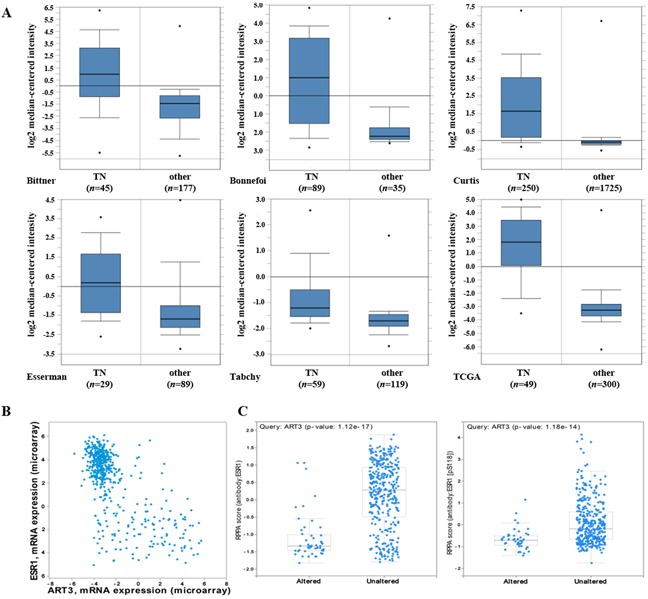
ART3 mRNA are highly expressed in basal-like/triple negative (TN) breast cancers **A.** ART3 mRNA levels were elevated in TNBCs analyzed using cDNA microarray datasets in the Oncomine database. **B.** Plots showed between ESR1 (Y axis) and ART3 (X axis) mRNA levels in all patients from the dataset used in Figure 1A. Data showed a negative correlation between ESR1 and ART3. **C.** Box plots showed high ART3 mRNA levels were correlated with low ESR1 protein (left) and pS118 ESR1 (right) levels in all patients analyzed using the dataset in Figure 1. Y axis: Protein and phosphoprotein data based on reverse phase protein array (RPPA). X axis: ART3 mRNA level status. The experimental results described above had statistical significance (P<0.05).

We first examined whether ART3 mRNA is expressed differentially in human breast cancer molecular subtypes using the publicly available Oncomine database (http://www.oncomine.org). As shown in Figure 1A, ART3 mRNA levels were higher in TNBC than other types in Bittner (GEO Accession No. GSE2109), Bonnefoi [18], Curtis [19], Esserman [20], Tabchy [21], and TCGA (http://tcga-data.nci.nih.gov/tcga/) datasets. Furthermore, we found the similar result in TCGA breast cancer dataset using online platform cBioPortal. ART3 mRNA was increased in nearly half of basal-like (49%) and very few HER2-amplified (5%) breast cancers, but not increased in any luminal A or B breast cancers. In the same TCGA dataset, we found the mRNA level of ART3 was negatively correlated with that of ESR1, which is a typical luminal marker (Figure 1B). Consistently, increased ART3 mRNA was correlated with decreased total and phosphorylation (pS118) of ESR1 protein (Figure 1C). These data indicated that ART3 might be a marker for TNBC.

### High ART3 levels are correlated with worse survival in breast cancer patients

**Figure 2:**
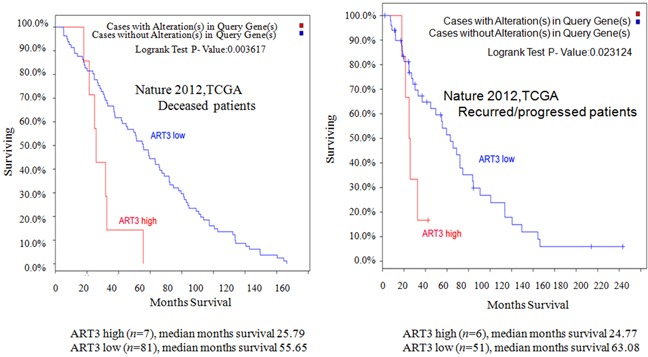
ART3 levels are correlated with shorter overall survival Kaplan–Meier survival curves for overall survival in deceased **A.** and recurred/progressed **B.** patients analyzed using The Cancer Genome Atlas (TCGA) Breast Invasive Carcinoma project (825 cases) dataset.

In order to further elucidate the clinical relevance of ART3 in breast cancer, we performed Kaplan-Meier survival analysis. As patients with basal-like breast cancer subtype have a higher rate of progression and worse survival, we focused on deceased patients and patients with tumor progression. We found that patients with high ART3 mRNA levels showed worse survival in both deceased (Figure 2A) and progressed (Figure 2B) group. Our data indicated that high levels of ART3 are correlated with worse prognosis in breast cancer.

### Expression of ART3 protein in human breast cancer tissues and cell lines

**Figure 3:**
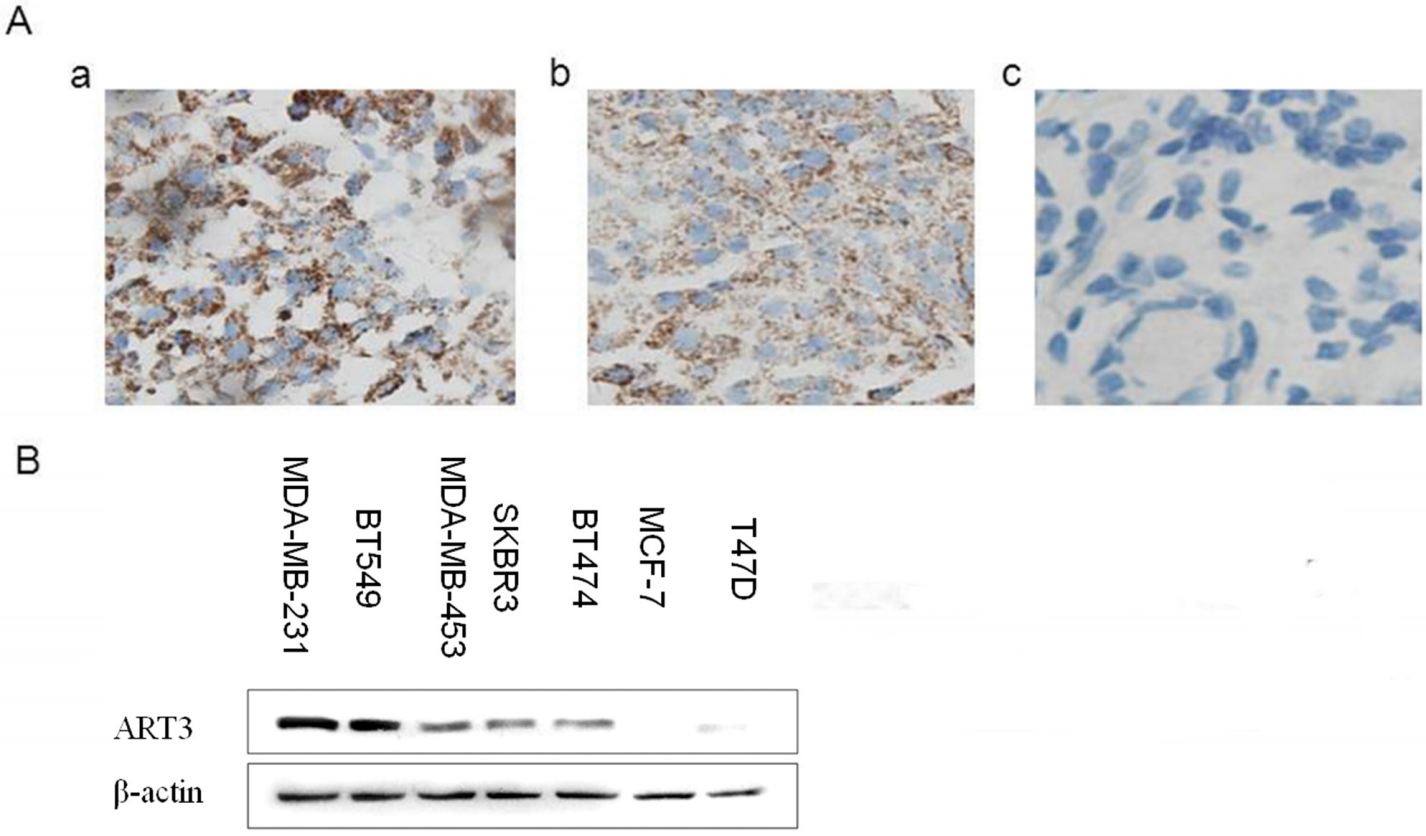
ART3 is overexpressed in triple-negative breast cancer **A.** Immunohistochemistry of ART3 in human breast cancers. (Magnification×400). Representative images of positive (a and b) and negative (c) ART3 staining are shown. **B.** ART3 expression in human breast cancer cell lines. By western blotting, ART3 expression was higher in TNBC MDA-MB-231 cell and BT549 than in other cells carrying at least one of hormone receptor. β-actin was used as a loading control.

The association of ART3 mRNA expression with basal-like breast cancers prompted us to study whether ART3 might be overexpressed in TNBC. Using a cohort of fixed breast cancer samples, immunohistochemistry detected 3 in 39 ER+ samples, 2 in 31 ER-/Her-2+ samples, and 28 in 43 TNBC samples were positive for ART3 expression. This indicated that, relative to the ER+ group or ER-/Her2+ group, there was a higher percentage of samples with ART3 positive expression in TNBC group. The difference between the groups was statistically significant (*χ*^2^=43.31, *P*<0.001) as evidenced by the *χ*^2^ test (Table 1, Figure 3A). To further address whether ART3 is correlated with TNBC breast cancers, we also investigated the expression of ART3 in a panel of 7 breast cancer cell lines including two TNBC (MDA-MB-231 and BT549), two ER-/Her2+ (SKBR3 and MDA-MB-453), one Her2+ (BT474), and two ER+/Her2- (MCF-7 and T47D) cell lines. We found that the two TNBC cell lines expressed higher ART3 compared to other five non-TNBC cell lines. Of note, in two ER+ breast cancer cell lines, ART3 expression were almost undetectable (Figure 3B). These data confirmed that ART3 was specifically expressed in TNBC and basal-like breast cancers, and also suggested that a cross-inhibitory mechanism might exist ART3 and ESR1.

**Table 1:** ART3 protein expression is associated with triple-negative breast cancer

	Case	ART3	
		+ (%)	− (%)
ER (−)/Her (+)	31	2 (6.45)	29 (93.55)
ER (+)	39	3 (7.69)	36 (92.31)
TNBC	43	28 (65.11)	15 (34.89)

### ART3 expression promotes TNBC cell proliferation

**Figure 4:**
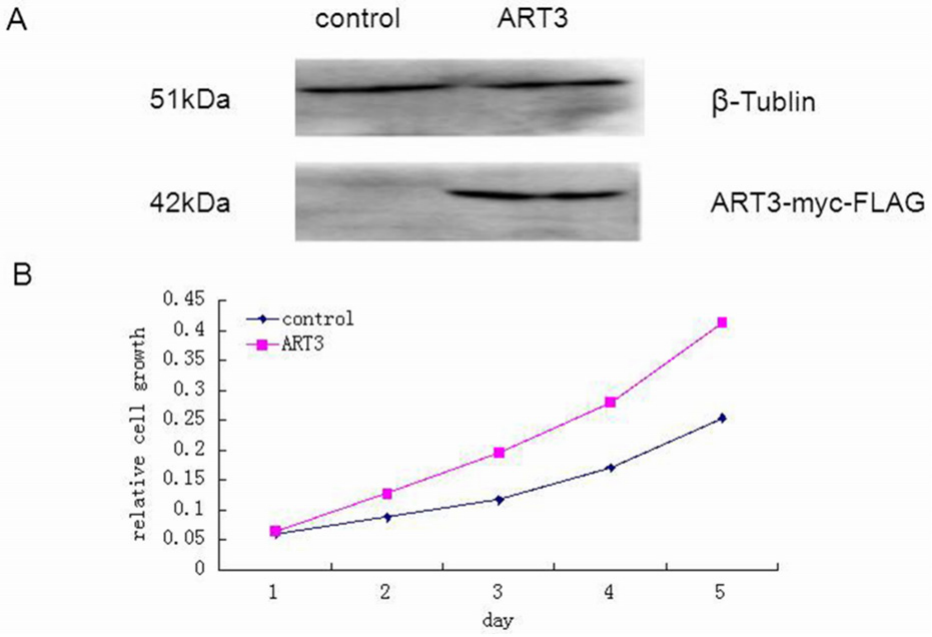
ART3 overexpression increases cell proliferation **A.** Western blotting analysis of ART3-myc-FLAG protein expression in control and stably transfected cells. **B.** Proliferation of control and ART3-overexpressing cells were measured by MTT assays. Cells were grown in normal culture medium.

**Figure 5:**
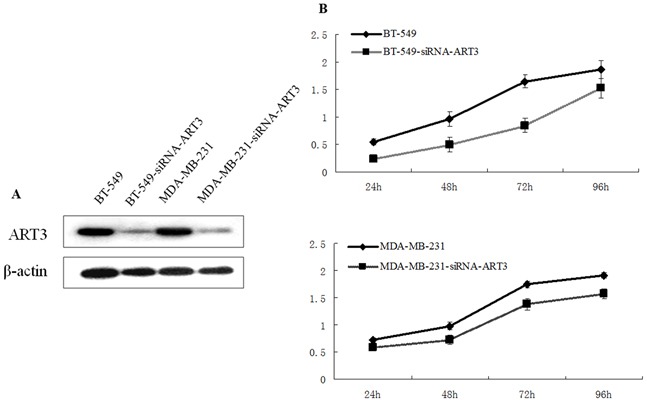
ART3 knockdown suppresses cell proliferation **A.** Western blotting analysis of ART3 expression in control and siRNA-ART3 cells. **B.** Proliferation of control and siRNA-ART3 cells was measured by MTT assays. Cells were grown in normal culture medium.

Next, we tested whether ART3 could increase the proliferation of TNBC cells. Using MDA-MB-231 cells overexpressing ART3, or MDA-MB-231 and BT549 cells transfected with either siRNA specifically to ART3 or MOCK-siRNA, we performed MTT assays. The results showed that ART3 overexpression significantly promoted MDA-MB-231 cell proliferation (*P*=0.021, Figure 4A and 4B). ART3 knockdown significantly suppressed the proliferation of MDA-MB-231 and BT549 cells (*P*=0.045, and 0.032, respectively, Figure 5A and 5B).

### ART3 overexpression suppresses cell apoptosis

**Figure 6:**
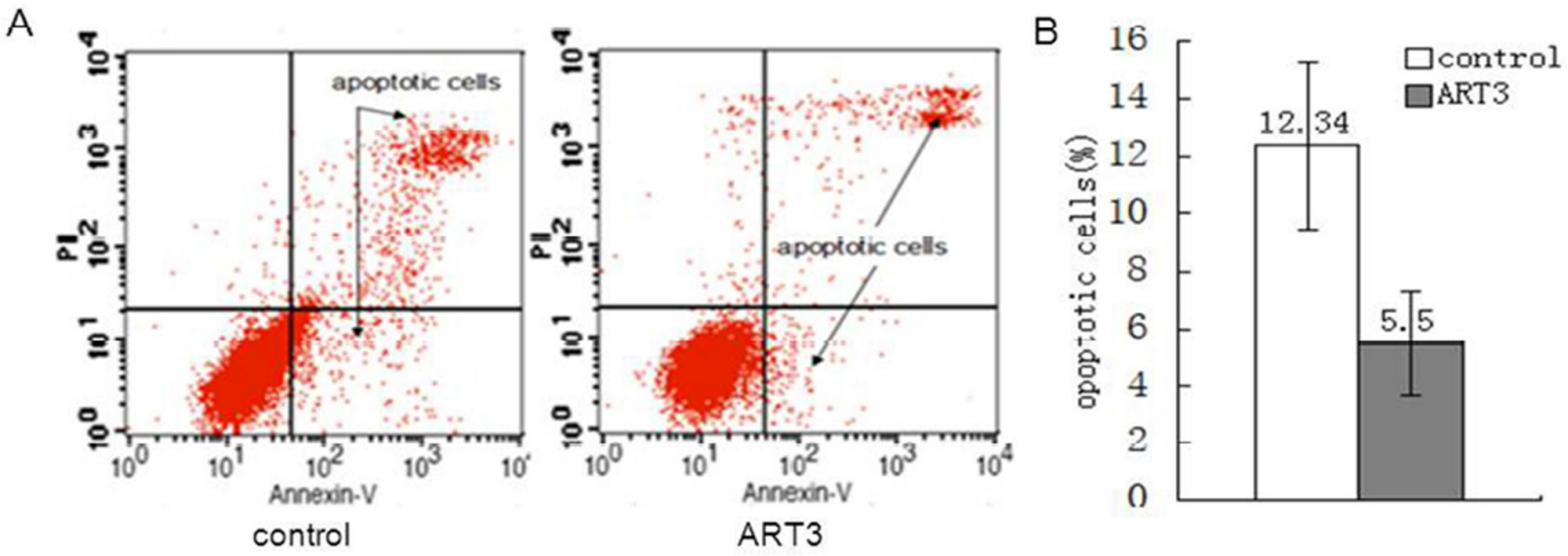
ART3 overexpression reduces breast cancer cell apoptosis **A.** Apoptotic rates of control and ART3-overexpressing cells were assessed by Annexin V and PI double staining followed by flow cytometry analysis. **B.** Data represent mean ± SD of three independent experiments. *P*<0.05.

We then performed flow cytometry assays to determine whether ART3 overexpression could decrease the apoptotic rate of MDA-MB-231 cells. Our control vector-transfected cells displayed a significantly higher apoptotic rate (12.34 ± 2.90%) than ART3-overexpressing MDA-MB-231 cells (5.50 ± 1.80%, P< 0.05; Figure 6). These results suggested that ART3 overexpression-induced cell growth might be partially resulted from reduced apoptosis.

### ART3 expression promotes cell invasion *in vitro*

**Figure 7:**
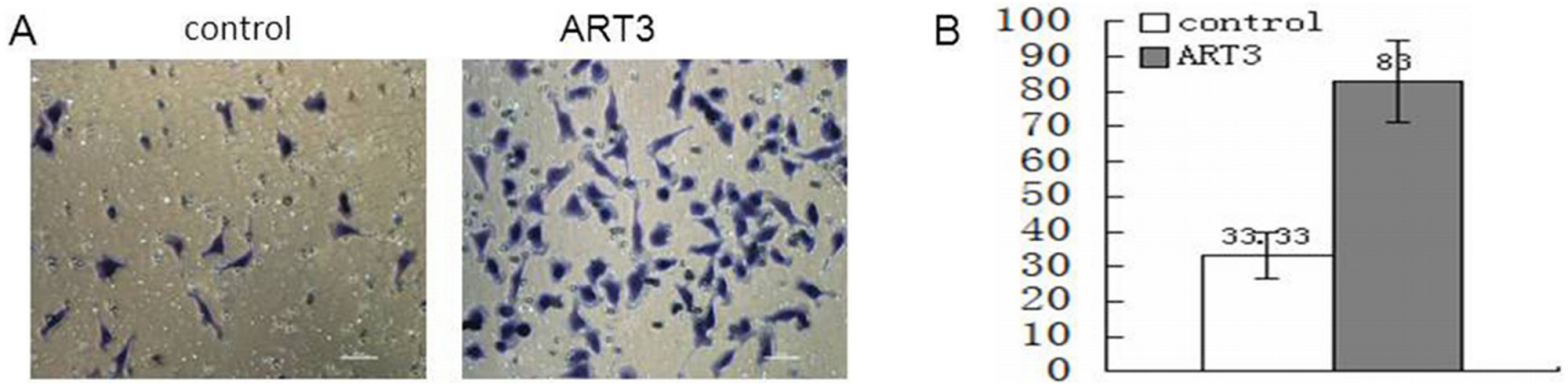
ART3 overexpression increases cell invasion **A.** Invasion of control and ART3-overexpressing cells were measured using transwell chamber assays (original magnification, ×200). **B.** Data represented average cell number from 5 viewing fields *P*<0.05.

**Figure 8:**
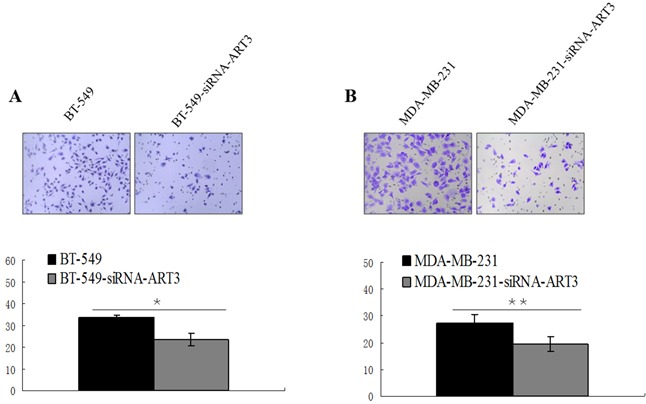
ART3 knockdown suppresses cell invasion Transwell chamber assay was used to measure the invasion ability of control and ART3-siRNA-cells, BT-549 **A.** and MDA-MB-231 **B.** cell lines. The original magnification was ×200, and data represent average cell number from 5 viewing fields *P*<0.05.

We next explored whether ART3 could stimulate the *in vitro* invasion of BT549cells and ART3-transfected MDA-MB-231 cells. Additionally, MDA-MB-231 and BT549 cells transfected with either siRNA of ART3, or MOCK-siRNA were also used to perform the modified trans-well assay. ART3 overexpression significantly stimulated MDA-MB-231 cell invasion compared with the control cells (*P*=0.014; Figure 7). ART3 knockdown suppressed the invasion ability of MDA-MB-231 and BT549 cells (*P*=0.011, and <0.001, respectively, Figure 8A and 8B).

### ART3 overexpression activates AKT and ERK in breast cancer cells

**Figure 9:**
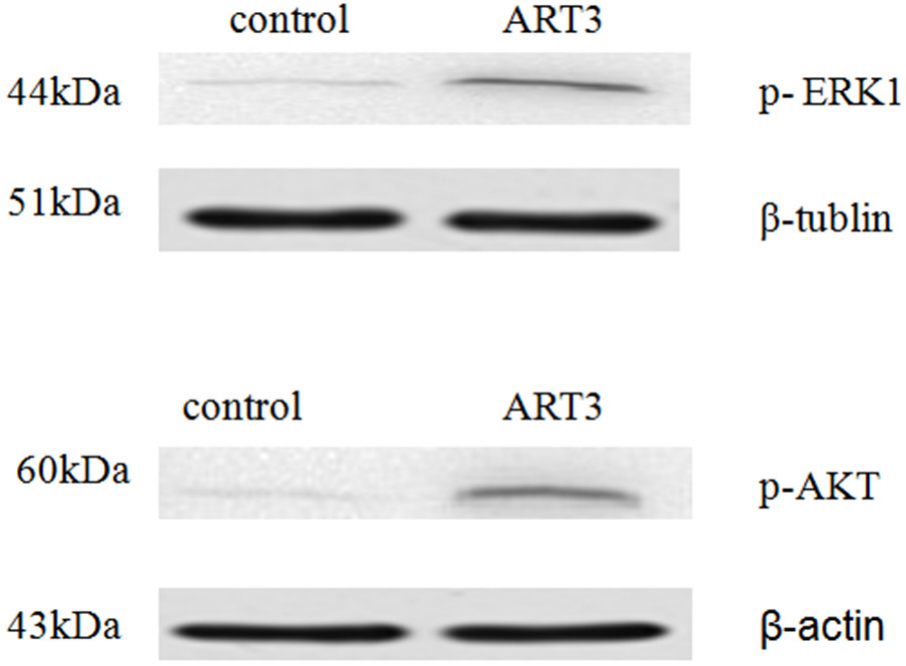
ART3 overexpression increases AKT and ERK (ERK1 was detected only) activation in breast cancer cells p-ERK and p-AKT levels in control and ART3-expressing cells were measured using western blotting.

**Figure 10:**
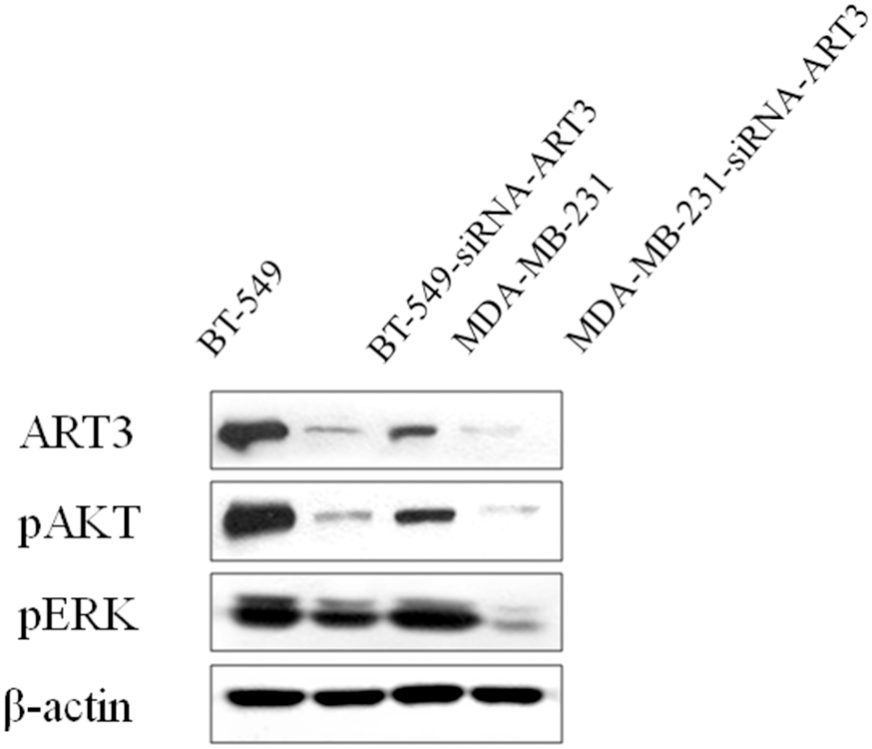
ART3 knockdown downregulates p-AKT and p-ERK Western blotting demonstrated that expression of both p-AKT and p-ERK were lower in AKT3-siRNA cells than the controls.

**Figure 11:**
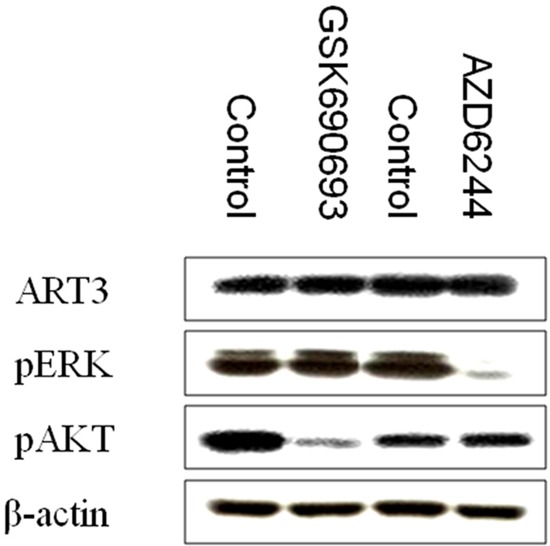
p-AKT and p-ERK inhibition does not influence ART3 expression Western blotting demonstrated that ART3 expression did not exhibit a significant change in MDA-MB-231 cells treated either p-AKT inhibitor (GSK690693) or p-ERK inhibitor (AZD6244).

To explore the potential mechanisms underlying the aggressive cell behavior induced by ART3 expression, two microarray datasets including TNBC patients were downloaded from the public database mentioned above. These patients were then divided into two groups according to the quartiles of ART3 expression levels: one group of patients with ART3 expression at upper quartile (P25), and the other group of patients with ART3 expression at lower quartile (P75). Then moderated *t*-test was performed to measure the differences of gene transcription between the control (P25) and the ART3-overexpression groups (P75). Numbers of differential expressed genes were found. After adjusting the selection criteria by decreasing the *P* value, no significantly enriched pathway was found by functional enrichment analysis. However, dysregulated expression of several genes in the downstream of the PI3K-AKT signaling pathway and the MAPK signaling pathway were observed (Table 2), most of the genes in this list were related to cell cycle or apoptosis. This result could, at least partially, explain why ART3 overexpression induced higher proliferation and less apoptosis in TNBC cells. This led us to examine whether ART3 could regulate ERK/MAPK and/or AKT kinases. We thus examined the activation of ERK1 and AKT in control cells and MDA-MB-231 cells with ART3 expression, and found that the expression levels of phosphorylated ERK1 (p-ERK1) and phosphorylated AKT (p-AKT) were higher in the ART3-transfected MDA-MB-231 cells (Figure 9). Knockdown of ART3 in both MDA-MB-231 or BT549 cells reduced the levels of phosphorylated ERK1/2 and AKT without altering the expression level of their total protein levels (Figure 10). In addition, we found that treatment of BT549 and MDA-MB-231 cells with MEK inhibitor AZD6244 or AKT inhibitor GSK690693 did not influence the ART3 expression, indicated that ART3 was not induced by ERK and AKT (Figure 11). ART3 might act upstream of AKT and ERK.

**Table 2:** Dysregulated expression of genes in the PI3K-AKT signaling pathway and MAPK signaling pathway

Symbols	GSE21653		GSE65194		GSE68115	
	logFC	adj.P.Val	logFC	adj.P.Val	logFC	adj.P.Val
p21	−	−	−0.405	0.033	−	−
CDK2	0.205	0.017	−	−	0.205	0.017
CDK6	0.754	<0.001	0.763	<0.001	0.754	<0.001
CCNE1	0.611	<0.001	0.851	<0.001	0.611	<0.001
CDKN1B	−0.390	0.009	−	−	−0.390	0.009
RBL2	−0.330	<0.001	−	−	−0.330	<0.001
FASLG	−	−	0.273	0.031	−	−
BAD	−0.166	0.023	−0.219	0.022	−0.166	0.023
MCL1	−	−	0.627	<0.001	−	−
STMN1	0.571	2.87E-11	0.635	<0.001	0.571	<0.001
cPLA2	1.357	<0.001	1.835	<0.001	1.357	<0.001
RSK2	0.369	0.005	0.708	<0.001	0.369	0.005
ELK-1	0.167	<0.001	0.231	<0.001	0.167	<0.001
Sapla	0.170	<0.001	0.406	<0.001	0.170	<0.001
c-myc	0.930	<0.001	2.090	<0.001	0.930	<0.001

*logFC and adjusted p values of significantly differential expressed genes were list in this table, otherwise, ‘-’ was instead.

### ART3 overexpression enhances mammary tumor growth in xenograft models

**Figure 12:**
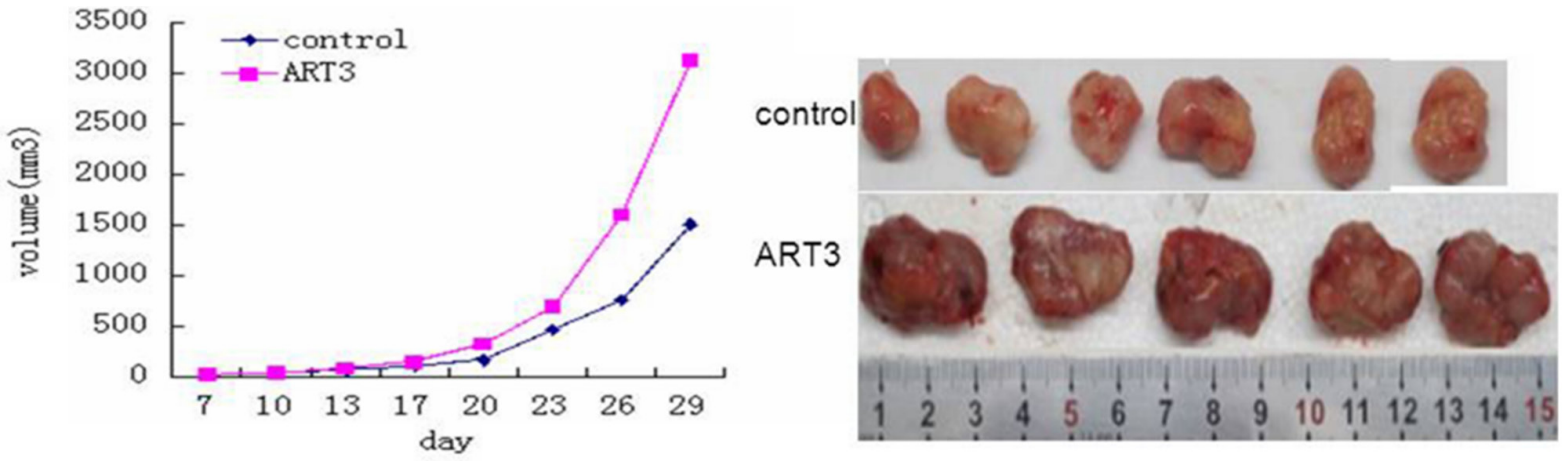
ART3 overexpression enhances mammary tumor growth in xenograft models Growth curves of mammary tumors after orthotropic injection of control and ART3-overexpressing MDA-MB-231 cells in nude mice. Data represented mean ± SD (n=8). P<0.05.

**Figure 13:**
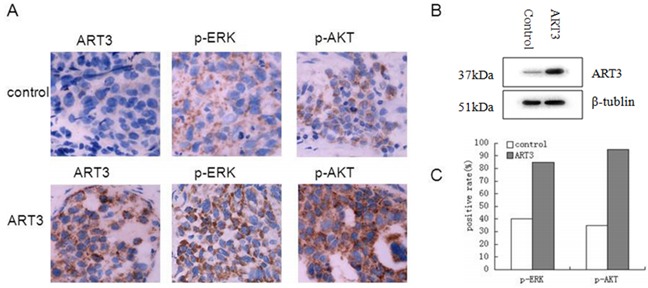
Detection of ART3, p-ERK, and p-AKT expression in xenograft tumors **A.** Immunohistochemistry of ART3, p-ERK and p-AKT both in control group and ART3 group (Manification,×400). **B.** The expression of ART3 in xenograft tumor was detected by western blotting. **C.** Score of p-ERK and p-AKT staining in (A). The percentages of positive cells were plotted.

To further evaluate the effect of ART3 overexpression on TNBC cells *in vivo*, we established xenograft tumor models for MDA-MB-231 cells with/without ART3 overexpression. The experimental animals were sacrificed 30 days after the xenografts reached the size of 3 mm x 3 mm (Figure 12), and xenograft tumors were used to measure the expression of either ART3 by immunoblotting or the expression of phosphorylated ERK1 and AKT by immunohistochemical staining. We found that the xenograft tumors of MDA-MB-231 cells with ART3 overexpression grew faster. Additionally, xenografts with higher level of ART3expressed higher levels of phosphorylated AKT and ERK1, when compared to xenografts of control MDA-MB-231 cells (Figure 13A, 13B and 13C). These data, consistent with *in vitro* studies, clearly demonstrated that ART3 might promote TNBC cell proliferation and apoptosis via mechanisms that involved activation of ERK and/or AKT.

## DISCUSSION

TNBC normally has the lowest five-year survival rates and disease-free survival rates, compared with other breast cancer types that overexpress ER+ and/or Her-2+. In this study, we found that ART3 displays the highest expression in human TNBC.

Protein post-translational modifications occur in the later stage of protein synthesis with critical functions. These modifications can cause alterations in physicochemical properties and spatial conformations, thereby influencing protein biological function. Recent studies have revealed their role in cell proliferation, invasion, apoptosis, signal transduction, DNA modification, and the immune response. Adenosine diphosphate (ADP) ribosylation is a critical process in protein post-translational modification, and is related to modification of chromosomal function, occurrence of tumors, apoptosis, and cell death [22, 23]. ART and ecto-ADP-ribosylprotease can catalyze the reversible process of ecto-ADP-ribosylation [24] and transfer ADP-ribose in NAD+ ecto-ribosyltransferase to specific amino acids in the target protein through ecto-ADP-transferase. In the study by Lodhi et al., ecto-ADP-ribosylation was found to be involved in the formation and secretion of apoptotic bodies in apoptosis [25]. Another study showed that ART1, another member in the ART family, can anchor phosphatidylinositol, which can regulate ADP-ribosyltransferase activity. ADP-ribosyltransferase can also inhibit the proliferation and differentiation of mice myoblasts [26].

To date, the role of ecto-ADP-ribosylation in cancer cell function has not been well defined. In this study, the TNBC cell line MDA-MB-231 was used for the stable transfection of a plasmid containing the ART3 gene, in order to explore the effects of ART3 on MDA-MB-231 cells proliferation, invasion, and metastasis. MDA-MB-231 cells with ART3 overexpression proliferated faster than the control group. We also showed that apoptosis rate was lower in the ART3 overexpression group than in the control group. Our data is in consistence with previous reports showing that ART may function in cell proliferation [3, 5]. Since PARP was reported to actively participate in DNA repair and genome stabilization [27], our discoveries suggest that the inhibitory effect of ART3 on cell apoptosis might be related to PARP.

MAPK is a Ser/Thr protein kinase, and its signal transduction pathway is critical in eukaryotes for the modulation of various biological processes, such as cell growth, development, division, and death. ERK belongs to the MAPK family, and is critical for cell growth, development, and division. The PI3K/AKT signal pathway can lead to malignant transformation in the cell, and also promotes the survival and proliferation of tumor cells. In addition, the PI3K/AKT pathway is related to migration, adherence, angiogenesis, and extracellular matrix degradation of tumor cells. We found that the expression of ART3 induces phosphorylated ERK and phosphorylated AKT in TNBC cells and xenograft tumors, suggesting that ERK and AKT may mediate the effects of ART3 in TNBC cell function. More work is need to uncover the mechanism underlying ART3 regulation of ERK and AKT activation. In conclusion, this study demonstrates the higher expression of ART3 in TNBC and implicates ART3 as a critical marker or TNBC. This study establishes the foundation for further exploration of the biological function and mechanism of ART3 in cancer cells.

## MATERIALS AND METHODS

### Materials

Human breast cancer specimens were supplied by the Department of Pathology in the Second Affiliated Hospital of Chongqing Medical University (Chongqing, China). The human breast cancer cell lines MDA-MB-231, BT-549, MCF-7, T47D, BT474, MDA-MB-453, and SKBR3 were originally purchased from the American Type Culture Collection (ATCC, Manassas, VA, USA) and kept in the Health Sciences College of Chongqing Medical University. All cells were grown in Roswell Park Memorial Institute 1640 (RPMI-1640) medium (Invitrogen, Carlsbad, CA, USA) with 10% FBS. All cells were maintained in a humidified atmosphere of 5% CO_2_ at 37°C. The pMCV-ART3-Myc-tag recombinant eukaryotic plasmid was purchased from Origene Biotech Company (Carlsbad, CA, USA). Lipofectamine 2000 and G418 were purchased from Invitrogen. Mouse anti-human beta-actin monoclonal antibody and horseradish peroxidase (HRP)-labeled goat anti-mouse IgG were purchased from Zhongshan Golden Bridge Biotechnology Co. Ltd (Beijing, China). ART3 antibody was purchased from Santa Cruz Biotechnology (Santa Cruz, CA, USA). Antibodies against phosphorylated protein kinase B (PKB)/AKT and the phosphorylated mitogen-activated protein kinase (MAPK) extracellular signal-related kinase ERK1 were purchased from Bioss Biotechnology Co. Ltd (Beijing, China), phospho-ERK1/2 (Thr202/Tyr204), ERK1/2 were purchased from Cell Signaling (Beverly, MA, USA). Beta-tubulin monoclonal antibody was purchased from Walterson Biotechnology Co (Liaoning, China). Matrigel was purchased from Sigma-Aldrich (Natick, MA, USA). Balb/c female mice of 6-8 weeks old were supplied by the Animal Experimental Center of Chongqing Medical University. AZD6244 and GSK690693 were obtained from Selleck Chemicals (Shanghai, China). All animal experiments were strictly adhered to local and federal regulations, and approved by the Experimental Animal Committee of Chongqing Medical University before initiation.

### Methods

#### Oncomine database analysis

ART3 mRNA expression in human breast cancer tissues was analyzed by searching the Oncomine database (www.oncomine.org) with keyword “ART3”, “breast cancer”, and “cancer vs. cancer” filter.

#### TCGA breast cancer database analysis

The TCGA dataset was analyzed in a free web-based platform, the cBioPortal for Cancer Genomics [16]. According to the PAM50 classification system, this dataset included 80 cases (9.7%) of basal-like, 235 (28.5%) cases of Luminal A, 131 cases (15.9%) of Luminal B, and 58 cases (7.0%) of Her2-amplified breast cancers. 727 cases (88.1%) were disease-free and 57 cases (6.9%) were recurred or progressed. 721 cases (87.4%) were alive and 88 cases (10.7%) were deceased. Protein and phosphoprotein data analysis was done in RPPA platform [17] using cBioPortal. Overall survival results were displayed as Kaplan-Meier plots and *P* values were calculated by Log-Rank test.

#### GEO breast cancer database analysis

Two microarray datasets employed in this study were publicly available at GEO (http://www.ncbi.nlm.nih.gov/gds/) of NCBI with the accession numbers: GSE21653 and GSE65194. The CEL files containing the raw data from each experiment were directly downloaded from the websites with particular accession numbers. These datasets were normalized via Robust Multi-Array Analysis (RMA). The normalized expression values (on a log-2 scale) of probes representing the same gene were averaged. Additionally, one normalized protein dataset (GSE68115) of TNBC was downloaded from GEO.

#### Immunohistochemical staining

Tumor samples of 113 breast cancer cases were randomly collected from the tumor-bank of the Department of Breast, Thyroid, and Pancreas in the Second Affiliated Hospital of Chongqing Medical University. These patients were enrolled for surgery from January 1st, 2008 to December 31st, 2012, including 39 ER (+), 31 ER (−)/HER-2(+), and 43 TNBC cases. Immunohistochemical (IHC) staining was used to detect the expression of ART3 in paraffin-embedded breast cancer tissue. Briefly, paraffin sections were sequentially de-waxed, hydrolyzed, and incubated in 3% H_2_O_2_ at room temperature for 20 min. Sections were washed with phosphate-buffered saline(PBS) and blocked with 5–10% goat serum for 30 min. After 18-24h incubation with primary antibody at 1:100 dilution in blocking buffer at 4°C, secondary antibody at 1: 3000 dilution in PBS with 1% BSA was added on breast tissue sections for 1h at room temperature. Sections were finally treated with HRP-labeled streptavidin, stained with 3,3′ diaminobenzidine (DAB), washed with distilled water, re-stained by haematoxylin, and mounted.

Five fields (200×) were observed in each section. The percentage of positive cells were scored as: 0 for <5%, 1 for 5%–25%, 2 for 26%–50%, 3 for 51%–75%, and 4 for 76%–100%. The staining intensity of cells was scored as: 0 for colorless, 1 for light yellow, 2 for brownish yellow, 3 for brown. The final score was calculated as the percentage score multiplied by intensity score (0 for negative (−), 1–4 for lightly positive (+), 5–8 for positive (++), and 9–12 for strongly positive (+++)).

#### Cell culture and treatments

The human breast cancer cell lines MDA-MB-231, BT-549, T47D, MCF-7, BT474, MDA-MB-453 and SKBR3 were cultured in RPMI1640 media with 10% fetal bovine serum (FBS) at 37°C in a 5% CO2 saturated incubator. MDA-MB-231 cells were treated with DMSO, 100nM GSK690693 (AKT inhibitor, Selleck Chemicals, Houston, TX, USA), or 100 nM AZD6244 (MEK inhibitor, Selleck Chemicals, Houston, TX, USA) for 24 hours before harvesting for protein expression analysis.

#### Detection of ART3 expression in different cell lines by western blotting

Cells were harvested by scraping. Protein was extracted following lysis with RIPA buffer. The protein concentration was detected by the bicinchoninic acid (BCA) assay. 50μg of protein were separated by 12% sodium dodecyl sulfate polyacrylamide gel electrophoresis (SDS-PAGE), and transferred to a 0.45-μm pore size polyvinyl difluoride membrane by the Semi-Dry. The membrane was blocked in fat-free milk for 1 h at room temperature. Beta-tubulin antibody and mouse primary antibody against ART3 were diluted to 1:1000. The primary antibody was added and incubated at 4°C overnight. The membrane was washed twice with 1× TBST for 10 min each, and then once with TBS for 10min. HRP-labeled rabbit anti-mouse antibody (1:1000 dilution) was added to the washed membrane and incubated for 1 h at room temperature. After three washes, enhanced chemiluminescence (ECL) reagent was used for imaging analysis.

#### Establishment of MDA-MB-231 cells stably overexpressing ART3

The pCMV-ART3-Myc-tag plasmid or empty vector was transfected into MDA-MB-231 human breast carcinoma cells by using the Lipofectamine 2000 reagent following the manufacturer's instructions. After two-week selection under 800μg/ml G418, single colonies were expanded via the limited dilution method in RPMI1640 containing 600μg/ml G418 for 2 weeks, and ART3-overexpressing sublines were screened by western blotting using the antibody against ART3.

#### Establishment of MDA-MB-231 cells and BT-549 cells with ART3 knockdown

WDR79 siRNA sequence (sense sequence 50-AATCAGCGCATCTACTTCGAT-30, antisense sequence 50-AAATCGAAGTAGATGCGCTGA-30), which had been proved to knock down WDR79 effectively, were purchased from GenePharma (Shanghai, China) WDR79 siRNA sequence (sense sequence 50-AATCAGCGCATC TACTTCGAT-30, antisense sequence 50-AAATCGAAG TAGATGCGCTGA-30), which had been proved to knock down WDR79 effectively, were purchased from GenePharma (Shanghai, China).

siRNAs for ART3 was synthesized by Shanghai GenePharmaCo.Ltd. with the following target sequences: 5′-GGCCAAUCUCGAGAAGAUU-3′. siRNAs were transfected at 100pmol/well into 96-well plate. Cells were seeded in plates 24 hours before transfection with siRNAs by Lipofectamine RNAiMAX according to the manufacturer's protocols. Cells were lysed at 48 hours after transfection. Protein expression was analyzed by western blotting.

#### Detection of cell proliferation by 3-(4,5-dimethylthiazol-2-yl)-2, 5-diphenyltetrazolium bromide (MTT) assay

96-well plates were used for the transfection group and control group, respectively. One control well without cells was set up in both groups. 3×10^3^ cells in 200μl RPMI1640 medium supplemented with10% FBS were inoculated to each well of 96-well plates. To measure cell growth, 20μl of 5mg/ml MTT solution was added into each well of cells with inoculation for 12, 24, 36, 48, and 72 h, and then incubated for 4h. DMSO (150 μl/well) was added to the medium, and the plates were shaken for 10 min in dark. The absorbance value in each well was detected at 492nm in the MTT enzyme-linked immunometric meter. The value for each group was calculated as the average absorbance subtracted by the absorbance value in the blank well. The cell growth curve was generated with time on the X-axis and the corrected absorbance value on the Y-axis. This experiment was repeated for three times.

#### Detection of cell invasion by transwell

A 50μg/ml Matrigel solution was diluted with serum-free medium in a 1:8 ratio, aliquoted to the upper chambers of the Transwell plate, and incubated at 37°C for 30 min. A 200μl serum-free medium containing 1×10^5^ cells was added to each upper chamber of the Transwell plate.500 μl of RPMI1640 media with 10% FBS was added in the lower chamber. After 24h incubation at 37°C, 5% CO_2_, cells without invasion were swapped and the filter was inversely placed for 10–15 min for drying. Then crystal violet (0.1%) was used to stain the cells for 30min at room temperature. The cells were washed twice with PBS and counted under a microscope at 200X magnification.

#### Detection of cell apoptosis by flow cytometry

Flow cytometry assay was performed to detect cell apoptosis by using Annexin V-FITC/PI kit (Molecular Probes, ThermoFisher, Cambridge, MA, USA) following the manufacture's protocol. Briefly, cells were digested, collected by centrifugation, and washed with 0°C PBS twice. The cells were re-suspended in 200μl buffer provided by Annexin-V reagent kit. Annexin-V-fluorescein-isothiocyanate (FITC; 10μl) was added to the cell suspension, gently mixed, and placed in 4°C for 30min in the dark. Subsequently, 300μl buffer and 5μl propidium iodide (PI) were added to the cell suspension and incubated for 5min. Cytometry was performed using the FACS Calibur (BD Biosciences, San Jose, CA, USA) and Cell Quest software. Four distinct cell populations were distinguishable: 1) the viable population (Annexin V and PI negative cells); 2) the early apoptotic population (Annexin V positive and PI negative cells); 3) the late apoptotic population (Annexin V and PI positive cells); and 4) the necrotic or lysed population (Annexin V negative and PI positive cells).

#### Detection of phosphorylated ERK and phosphorylated AKT

Cells were harvested in 80-90 % confluency, lysed with ice cold RIPA buffer (plus phosphatase inhibitors). The protein concentration was detected by the bicinchoninic acid (BCA) assay. Cells extracts with 50 μl protein were loaded into each well and separated by 12% SDS-PAGE, and then transferred to a 0.45μm polyvinyl difluoride membrane by the Semi-Dry method. The membrane was blocked in TBST (Tris-buffered saline containing 0.05% Tween 20) plus 5% BSA for 1h at room temperature. Then the blots were incubated with antibodies against pERK, pAKT, ART3 (1:1000) and β-tubulin (1:1000) at 4°C overnight. Each of the membrane was washed twice with 1×TBST for 10 min and once with TBS for 10 min. HRPO-labeled rabbit anti-mouse antibody (1:500) was added to the washed membrane and incubated for 1h at room temperature. After three washes, enhanced chemiluminescence (ECL) reagent was used for visualization.

#### Establishment of the xenograft tumor model

All animal experiments strictly adhered to local and federal regulations, and were approved by the Experimental Animal Committee of Chongqing Medical University before initiation. Balb/c nude mice (purchased from Southwestern Animal Center, Chengdu, China) weighing from 20 to 30g were inoculated subcutaneously with 1×10^6^cells of either ART3-overexpressing or normal MDA-MB-231 cells suspended in PBS to the No.4 mammary glands. Each group contained 8 mice. The growth of the xenografted tumor was measured once every 2–3 days, and the tumor volume was calculated by: Tumor volume (V, CM3) = ½× L×D^2^ (L is the long diameter of tumor and D is the short diameter of tumor). The tumor growth was plotted. The mice were sacrificed after 30 days since inoculation and the tumor tissues were harvested, aliquoted for subsequent experiments including western blotting and IHC staining to detect the expression of ART3, phosphorylated ERK, and phosphorylated AKT.

#### Statistical analysis

SPSS 17.0 software was applied for data analysis. The *χ*^2^ test was used to estimate the ART3 expressions among different human breast carcinoma cells. The *t* test was used in rest experiments. The data were represented as mean± standard deviation (SD), and statistical significance was indicated by *P*< 0.05.
